# The Multiple Roles of Microrna-223 in Regulating Bone Metabolism

**DOI:** 10.3390/molecules201019433

**Published:** 2015-10-26

**Authors:** Yong Xie, Lihai Zhang, Yanpan Gao, Wei Ge, Peifu Tang

**Affiliations:** 1 Department of Orthopedics, General Hospital of Chinese PLA, Beijing 100853, China; E-Mails: yong.xie301@gmail.com (Y.X.); zhanglihai@gmail.com (L.Z.); 2 National Key Laboratory of Medical Molecular Biology, Department of Immunology, Institute of Basic Medical Sciences, Chinese Academy of Medical Sciences, Beijing 100005, China; E-Mail: gaoyanpan@gmail.com

**Keywords:** miR-223, osteoclast, osteoblast, bone metabolism

## Abstract

Bone metabolism is a lifelong process for maintaining skeletal system homeostasis, which is regulated by bone-resorbing osteoclasts and bone-forming osteoblasts. Aberrant differentiation of osteoclasts and osteoblasts leads to imbalanced bone metabolism, resulting in ossification and osteolysis diseases. MicroRNAs (miRNAs) are pivotal factors in regulating bone metabolism via post-transcriptional inhibition of target genes. Recent studies have revealed that miR-223 exerts multiple effects on bone metabolism, especially in the processes of osteoclast and osteoblasts differentiation. In this review, we highlight the roles of miR-223 during the processes of osteoclast and osteoblast differentiation, as well as the potential clinical applications of miR-223 in bone metabolism disorders.

## 1. Introduction

Microscopic damage to the structure of bone is repaired by bone metabolism. This begins in the fetus and continues throughout life. It is essential in order to maintain the structure and mechanical strength of bone [1]. In bone metabolism, the destructive process of bone resorption is followed by the productive process of bone formation [2]. Osteoclasts are unique amongst somatic cells in that they can resorb bone matrices, and are the principal mediator of bone resorption [3]. Along with macrophages, they are derived from hematopoietic stem cells [4]. In contrast, osteoblasts and their precursors are, as with chondrocytes and adipocytes, of mesenchymal stem cell origin [5]. The interaction between osteoclasts and osteoblasts contributes to coupling of bone resorption and formation. Under normal circumstances, bone formation and resorption are in balance, as a consequence of precisely regulated processes [6]. The coordinated formation of osteoclasts and osteoblasts is critical for maintaining physiological bone metabolism [7], which is always determined by the differentiation of osteoclast and osteoblast precursors to form mature cells [8]. Abnormal bone metabolism, caused by enhanced osteoclast differentiation or decreased osteoblast differentiation, leads to a number of pathological bone destruction diseases [9], including rheumatoid arthritis (RA) and osteoporosis [10]. Such disorders are associated with a significant decrease in the patient’s quality of life [11] as well as a financial huge cost to healthcare systems [12].

To address these issues, a number of studies have focused on the differentiation of osteoclasts and osteoblasts to identify new therapeutic strategies for bone metabolism disorders. Specific factors expressed sequentially, and acting as an intracellular cascade, control the differentiation of hematopoietic progenitor cells into fully functioning osteoclasts [13,14]. These include macrophage-colony stimulating factor (M-CSF) and receptor activator of nuclear factor κB ligand (RANKL) [15]. These activate various intracellular signaling pathways and, in so doing, regulate the transcription and expression of osteoclast-specific genes [3]. Members of the fibroblast growth factor (FGFs) [16] and transforming growth factor beta (TGFβ) families are known to be essential for osteoblast differentiation [17]*.* The functions of these factors are regulated by microRNAs (miRNAs), a class of conserved RNAs [18], which function as post-transcriptional factors [19] in osteoclast and osteoblasts differentiation [20].

MiRNAs are small endogenous non-coding RNAs, made up of 22 nucleotides. They are well-studied and are known to affect the stability and expression of messenger RNA (mRNA) [21]. MiRNA genes are transcribed by RNA polymerase II to form primary miRNA (pri-miRNA) [22]. Nuclear pri-miRNA is processed to precursor miRNA (pre-miRNA), comprising 70 nucleotides, through the action of the RNase III enzyme, Drosha, and is exported from the nucleus [23]. Within the cytoplasm, the enzyme known as Dicer converts pre-miRNA into miRNA [24], which is loaded into the RNA-induced silencing complex (RISC) [25]. The miRNA is responsible for ushering RISC to the 3ʹ-untranslated region (3′-UTR) of the target mRNA. Nucleotides 2–8 are crucial for this process and are called the seed region [26]. The binding complementarity of the miRNAs to their seed region is usually imperfect [26]. Mechanisms known to be involved in the silencing of expression by the association of miRNAs with their target mRNA including the inhibition of the initiation, continuation of translation and the destabilization of target mRNA [21]. The latest miRBase release (v21, June 2014) contained 28645 miRNA loci that can produce 35828 mature miRNA products in 223 species [27]. One of these miRNAs, miR-223, which was first identified in the haematopoietic cells [28], is known to be highly conserved [29], and has been found in 10 species (miRBase release21, June 2014). It is reported that miR-223 is also expressed in bone, adipose tissue [30] and cardiac muscle tissue [31] and its effect on cell differentiation, inflammation and cancer is well studied [32].

Recent reports have demonstrated regulatory roles for miR-223 in bone metabolism [33,34,35], mainly in mediating repression of the critical proteins required for osteoclast and osteoblast differentiation. To provide a better understanding of the mechanisms by which miR-223 regulates bone metabolism, we highlight the roles of miR-223 in the repression of key factors during the processes of osteoclast and osteoblast differentiation, as well as the potential clinical applications of miR-223 in bone metabolism disorders.

## 2. Targets of miR-223 Involved in Bone Metabolism

The latest release of TargetScan (v7.0, Whitehead Institute for Biomedical Research, Cambridge, MA, USA August 2015, http://www.targetscan.org/vert_70/) [36] predicts 412 transcripts with conserved sites as biological targets of miR-223 in human and 284 transcripts in mouse (v6.2, Whitehead Institute for Biomedical Research, Cambridge, MA, USA, June 2012, http://www.targetscan.org/mmu_61/). Context++ scores of the sites are used to determine the predicted efficacy of targeting. They are measured using the contribution of 14 features, such as site-type, supplementary pairing and minimum distance [37]. The predictions are then ranked. According to these predictions, more than 20 miR-223 targets have been validated in studies conducted in humans or mice. Such targets include CCAAT/enhancer-binding protein-β (C/EBPβ), inhibitor of nuclear factor κB kinase subunit-α (IKKα), nuclear factor 1 A-type (NFIA) and fibroblast growth factor receptor 2 (FGFR2) [32].

FGFR2 and NFIA have been identified as miR-223 targets that participate in the osteoblasts differentiation and osteoclastogenesis [38], a process which includes osteoclast differentiation [35]. IKKα, a critical regulator of the NF-κB pathway, has been reported as a miR-223 target, with binding resulting in suppression of monocyte and macrophage differentiation [39]. Given that osteoclasts are monocyte-and macrophage-derived cells [40], IKKα is implicated in the mechanism by which miR-223 regulates osteoclast differentiation.

## 3. Regulation of miR-223 in Osteoclast Differentiation

### 3.1. MIR-223 Regulates Osteoclast Differentiation by Inhibiting NFIA Expression

NFIA is a CCAAT-box binding transcription factor, belonging to a dimeric DNA-binding nuclear factor I (NFI) protein family [41]. Zardo *et al*. demonstrated that miR-223 binds to specific sites within the promoter of its target gene NFIA and represses transcription by influencing epigenetic events [42]. Based on these reports, researchers investigated the potential of miR-223 to regulate osteoclast differentiation by inhibiting NFIA expression. Li *et al*. showed that miR-223 expression was down- regulated in the synovium of mice following intraperitoneal injection of a lentiviral vectors expressing the miR-223 target sequence (LVmiR-223T), with a concomitant increase in NFIA expression. Staining with the osteoclast-specific marker, tartrate-resistant acid phosphatase (TRAP), showed that osteoclast numbers were greatly reduced in LVmiR-223T-transduced bone marrow macrophages (BMMs), and increased NFIA expression was also detected. Similar results were also obtained using RAW 264.7 cells [33]. Hruska *et al*. found that in cells which expressed DGCR8, Dicer 1 or Ago and in Dicer-null osteoclast precursors (BMMs), NFIA was upregulated. However there was no detectable expression of NFIA in scrambled cells which expressed siRNA or in miR-223 expressing Dicer^wt/wt^ osteoclasts [43]. Subsequently, osteoclast formation assays using antisense miR-223 oligonucleotides showed that 92% inhibition of miR-223 induced down-regulation of TRAP-positive osteoclast formation in RAW264.7 cells compared with controls, while NFIA levels were upregulated. Previously, the same authors had found that in pre-miR-223 siRNA-expressing RAW264.7 cells in which the miR-223 was decreased by 66%, TRAP-positive osteoclast formation was unaffected [44], with no alterations of NFIA expression associated with this level of miR-223 inhibition. The authors speculated that the direct suppression of mature miRNAs by antisense oligonucleotides may be more effective than that of pre-miR-223 mediated by siRNA containing the stem-loop structure target sequence [43].

In turn, miR-223 expression is regulated by the competition between NFIA and CCAAT/enhancer-binding protein-α (C/EBPα) [45], which is known to function as an activator of target genes through binding directly to the consensus DNA sequence [46]. In undifferentiated NB4 cells, an acute promyelocytic leukemia cell line, NFIA binds the miR-223 promoter, maintaining miR-223 expression levels and consequently, low levels of miR-223-mediated translational repression. The process of differentiation involves NFIA being replaced by C/EBP α on the miR-223 promoter. This increases the expression of miR-223 [38], causing increased miR-223-dependant repression of NFIA mRNA and decreased NFIA protein expression [32]. In this way, NFIA limits miR-223 expression under undifferentiated conditions, while miR-223 limits NFIA expression during the differentiation process. In terms of miRNAs that inhibit mRNA translation [47], Shibuya *et al*. examined the NFIA expression in peripheral blood mononuclear cells at both the mRNA and protein levels 3 days after transfection with double-stranded-miR-223 and a double-stranded negative control [34]. It was found that miR-223 overexpression resulted in downregulated NFIA expression at the protein level but not at the mRNA level during osteoclast differentiation. In this way, NFIA will be upregulated when miR-223 expression is extremely low, thus, blocking osteoclast differentiation.

Moreover, Hruska *et al*. revealed a positive feedback loop between PU.1, macrophage colony-stimulating factor receptor (M-CSFR), NFIA and miR-223, which was involved in osteoclast differentiation [43]. In osteoclast precursors, PU.1, known as a transcription factor encoded by the SPI1 gene [48], is induced by M-CSF stimulated production of pri-miR-223. Pre-miR223 is processed by RNase III enzymes including Dicer, into mature miR-223. The result of this is downregulation of NFIA levels necessary for upregulation of M-CSFR levels in cells. Consequently, there is an increase in the expression of PU.1, MITF, and other transcription factors induced by M-CSF. As a consequence, cells differentiate into activated osteoclasts with upregulated expression of osteoclast-specific markers [43]. Furthermore, NFIA overexpression was shown to decrease osteoclast differentiation with downregulation of M-CSFR levels, while forced M-CSFR expression rescued osteoclast differentiation with upregulation of PU.1 levels in MCSF-dependent BMMs prepared from miR-223 Dicer-deficient mice. In accordance with this theory, M’Baya-Moutoula *et al*. confirmed that anti-miR-223 treatment inhibited osteoclastogenesis and overexpression miR-223 triggered differentiation in both RAW 264.7 cells and peripheral blood mononuclear cells (PBMCs) [49]. Although NFIA expression was found to be inversely associated with M-CSFR [33] and two putative NFIA-binding sites on the M-CSFR promoter, the precise identity of the cells expressing NFIA and the M-CSFR and the mechanism by which NFIA exerts negative effects on M-CSFR expression were not identified [43].

In conflict with the PU.1/miR-223/NFIA positive feedback theory, miR-223 overexpression was reported to block osteoclast differentiation in RAW264.7 cells [44] and PBMCs [34]. The mechanisms underlying these dual effects of miR-223 on osteoclast differentiation are not well-defined [50] and the precise interaction between NFIA and M-CSFR is likely to be critical for fully understanding this discrepancy. Given that the regulatory effect of NFIA on M-CSFR does not sufficiently explain why either overexpression or knockdown of miR-223 inhibits osteoclastogenesis, it can be speculated that there are other intracellular pathways which interact independently with NFIA in the regulation of miR-223 in osteoclast differentiation.

### 3.2. IKKα Could be Involved in the Regulation of miR-223 for Osteoclast Differentiation via Non-Canonical NF-κB Pathway

Shibuya *et al*. investigated the effect of over-expression miR-223 in osteoclastogenesis induced in vitro by RANKL. The results show that numbers of TRAP-positive osteoclasts were significantly reduced, suggesting that signaling pathways downstream of RANKL are the targets of miR-223 [34]. RANKL is expressed in osteoclast precursors and mature osteoclast [51]. The binding of RANKL and RANK is known to be pivotal in the regulation of mature osteoclast differentiation by activating intracellular signals, such as NF-κB [52]. IKKα, one of critical factors in the NF-κB pathway, is considered to be a target of miR-223 for inhibiting differentiation of osteoclasts [40], therefore, we focus here, on the possible roles of IKKα in the regulation of miR-223 for osteoclast differentiation via NF-κB pathway [53].

IKKα mRNA was predicted as target sequence of miR-223 by the Memorial Sloan Kettering Cancer Center miRNA database [54], and further identified in a series of experiments [39]. IKK is a part of the complex responsible for the induction of phosphorylation and the degradation of IκB-α in the conventional NF-κB pathway [55]. Therefore, IKKα also participates in the non-canonical NF-κB pathway [56]. MiR-223 target sites have been shown to contribute to the suppression of expression IKKα but did not affect the expression of IKKβ or IKKγ in monocytes or macrophages, suggesting that miR-223 specifically regulates the non-canonical NF-κB pathway but not the canonical NF-κB pathway [39].

NF-κB p100 is a non-canonical inhibitory κB protein. In unstimulated cells, it binds to RelB, preventing its translocation to the nucleus [57]. The ubiquitination and lysosomal degradation of TRAF3 is induced by RANKL by the action of TRAF2/cellular inhibitor of apoptosis 1/2 (cIAP1/2). This releases NF-κB-inducing kinase (NIK), enabling it to phosphorylate IKK-α. The effect of this is conversion of p100 to p52 by proteasomes [58]. Thus, miR-223 overexpression would downregulate p52, and miR-223 knockdown would upregulate p52 expression. Li *et al*. suggested two probable roles for p52 during the miR-223 regulated differentiation process [39]. The first is the prevention of the hyperactivation of new macrophages. The second is activation of gene transcription. The first role may be mediated, at least partially, by the upregulation of p52 in the absence of Re1B expression, leading to reduced gene transcription. The second role is likely to be a consequence of p52 binding to newly synthesized Re1B protein [59]. RelB has been implicated as a key factor in the activation of non-canonical NF-κB dimers that is detected at low levels in unstimulated macrophages [60]. RelB/p52 heterodimers can translocate to the nucleus to induce nuclear factor of activated T-cells, cytoplasmic 1 (NFATc1) [61], which is necessary for osteoclast differentiation [62]. Thus, it can be speculated that miR-223 knockdown leading to increased IKKα expression can inhibit osteoclast differentiation via excess p52 in the absence of RelB. The decrease in IKKα expression which is downregulated by miR-223 overexpression will also inhibit osteoclast differentiation with the reduction of RelB/p52 heterodimers.

Therefore, the non-canonical NF-κB pathway induced by binding of RANKL is probably involved in the regulation of miR-223 which down-regulates the expression of IKKα in osteoclasts differentiation. Although the precise mechanism remains to be confirmed in further experiments, this could be one explanation for the dual role of miR-223 in regulation of osteoclast differentiation.

## 4. The Regulation of miR-223 in Osteoblast Differentiation

The reciprocal regulation of the differentiation of adipocytes and osteoclasts involves miR-223. Guan *et al*. found that, following osteogenic treatment, miR-223 was reduced in preosteoblast MC3T3-E1. If miR-223 levels are supplemented by synthetic mimics, the growth of C3H10T1/2 and ST2 cells is slowed, whilst the differentiation of progenitor cells into adipocytes is induced, as is C/EBPα. Another illustration of the role of miR-223 on the formation of adipocytes from ST2 cells is provided by the overexpression of miR-223 caused by the lentivirus. The differentiation of ST2 cells into osteoblasts is prevented by miR-223 supplementation.

Dual luciferase reporter assay revealed that fibroblast growth factor receptor 2 (FGFR2) is a direct target of miR-223 [63]. FGFR2 is a critical regulator of osteoblasts. FGFR2 knockdown in C3H10T1/2 cells downregulated ERK phosphorylation, upregulated C/EBPα expression and dramatically enhanced the differentiation of the cells into adipocytes [35]. Other studies showed that activation of FGFR2 signaling enhanced osteoblast differentiation [17] by increasing runt-related transcription factor 2 (RUNX2) phosphorylation [64] mediated by extracellular signal regulated kinase (ERK) involved in MAPK pathway [65]. ERK also reduces C/EBPα activity by phosphorylation [66] and since C/EBPα induces miR-223 expression through binding to the miR-223 promoter sites [67], it is proposed that miR-223 down-regulates osteoblast differentiation through a C/EBPα/miR-223/FGFR2 regulatory feedback loop [35].

## 5. The Mediating Role of PU.1 in Regulation of miR-223 During Osteoblast and Osteoclast Differentiation

PU.1 is a member of the Ets family of transcription factors. It is important in the development of hematopoietic cell lines. This is particularly so for monocyte/dendritic cells [68]. It also has a role in osteoclast-specific gene expression with NFATc1 [69]. The regulation of specific genes in response to M-CSF and RANKL signaling during osteoclast differentiation is controlled by the transcription factors NFATc1, PU.1 and by microphthalmia-associated transcription factor (MITF), acting together [70].

As shown in previous studies, miR-223 overexpression induced by excess PU.1 would indirectly down-regulate the differentiation of osteoclasts and osteoblasts in aberrant bone metabolism. In these processes, the promoter of PU.1 is activated by C/EBPα [71], which cooperates with PU.1 and induces the expression of miR-223 [72]. In cells of the hematopoietic myeloid-osteoclast lineage, M-CSF induces PU.1 expression through M-CSFR [73,74]. Therefore, PU.1 acts as a mediating factor in the regulation of miR-223 in osteoclast and osteoblast differentiation.

**Figure 1:**
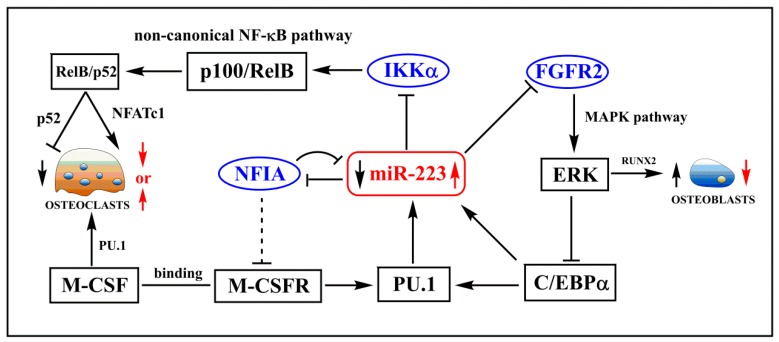
Schematic showing multiple regulatory networks of aberrant expression of miR-223 in regulating bone metabolism. MiR-223(shown as red) can regulate differentiation of osteoclasts and osteoblasts by repressing transcriptional factors, such as NFIA, IKKα and FGFR2 (shown as blue). Solid arrows indicate the promotion of cellular processes. Dotted lines indicate the mechanism has not been fully elucidated. Red short arrows indicate the regulation of overexpression miR-223, and black short arrows indicate the regulation of knockdown expression of miR-223.

In light of these findings, it can be concluded that regulatory networks involving miR-223 play multiple roles in regulating bone metabolism (Figure 1).

## 6. MiR-223 in Diseases with Bone Disorders

It has been found that miR-223 expression in synovium of patients with RA (21.0 ± 14.4-fold) is significantly higher than in the synovium of patients with osteoarthritis (OA) and healthy individuals [34]. Furthermore, the synovial tissue expressing high miR-223 exhibited poorly controlled bone destruction and severe acute synovitis. Increased levels of miR-223 expression detected in the ankle joints of mice during the collagen-induced arthritis (CIA) are accompanied by reduced NFIA levels and elevated M-CSFR levels. In this model, the level of miR-223 expression increased 29-fold on day 21 after collagen immunization (onset of arthritis), but had decreased to nearly basal level on day 42 (arthritic stage) [33]. Mild osteopetrosis in mice is known to be associated with high levels of miR-223 in osteoclasts [43].

Zhang *et al.* reported significantly lower miR-223 levels in osteosarcoma tissues compared with those in non-cancerous bone tissues, which is consistent with the previous studies conducted by Li *et al.* [75] and Xu *et al*. [76]. More significantly, they found that aggressive features, such as high tumor grade, metastases and recurrence were associated with the deregulation of the miR-223/ECT2 axis. Moreover, it also predicted a poor response to chemotherapy and decreased survival of patients with osteosarcomas [77]. The oncogenic heat shock protein 90B1 (HSP90B1) is a target of miRNA-223 in human osteosarcoma [75]. Furthermore, recent data show that miR-223 is abundantly expressed in multiple myeloma, its expression being the 15th highest among 722 miRNAs [78]. Extramedullary plasmacytomas can be distinguished from multiple myeloma on the basis of a lack of miR-223 [79].

Vascular calcification is found to be an actively process, which shares all features with bone metabolism [80,81]. Massy *et al*. found miR-223 upregulated in vascular smooth muscle cells (VSMCs) under inorganic phosphate conditions and promoting VSMC proliferation and migration by suppressing the expression of MEF2C and RHOB [31,82]. Studies showed that miR-223 was also increased in calcified aortas isolated from murine models of chronic kidney disease, which is implicated in vascular calcification [83,84]. M'Baya-Moutoula *et al*. identified that miR-223 induced osteoclastogenesis by affecting the expression of NFIA and RHOB and they demonstrated an approach that overexpresses miR-223 to selectively increase osteoclast-like activity in calcified vessels of chronic kidney disease-mineral and bone disorder (CKD-MBD) to alleviate vascular calcification [49].

All these findings demonstrated that miR-223 is an important regulator in diseases with bone disorders, with changes in its expression associated with the development stages and control of clinical signs (Table 1).

**Table 1:** MiR-223 in diseases with bone disorders.

Disease	MiR-223 Expression	Target Gene	Species	MiR-223 Effect	Reference
Rheumatoid arthritis (RA)	↑ (21.0 ± 14.4-fold)	NFIA	Human	Accompany by acutely severe synovitis and poorly controlled bone destruction	[33,34,43]
Osteoarthritis (OA)	↑ (4.1 ± 3.1-fold)	NFIA	Human	Not mentioned	[33,34,43]
Collagen-induced arthritis (CIA)	↑ (29-fold) on day 21 after collagen immunization	NFIA	Mouse	Accompany with reduction of NFIA and elevation of M-CSFR	[33,43]
Osteopetrosis	↓	NFIA	Mouse	Down-regulation of osteoclast differentiation	[43]
Osteosarcoma	↓	ECT2 HSP90B1	Human	Correlated with high tumor grade, the presence of tumor metastasis and recurrence, and the poor response to chemotherapy and also predicted a decreased survival prediction of patients	[75,76,77,85]
Multiple myeloma	↑		Human	Not mentioned	[78,79]
Vascular calcification	↑	RHOB, MEF2C	Human	Promotion of VSMC proliferation and migration	[81,86]
Chronic kidney disease-mineral and bone disorder (CKD-MBD)	↓	NFIA RHOB	Human	Obliterate the inhibitory effects of inorganic phosphate on osteoclastogenesis	[49]

## 7. Discussion and Perspectives

Here, a new aspect regarding the multiple roles of miR-223 in regulating bone metabolism has been revealed. There are at least two different pathways involved in the regulation of miR-223 in osteoclast differentiation, which exert antagonistic or synergistic functions at different expression levels. When miR-223 is elevated during abnormal bone metabolism, the expression of IKKα and NFIA are both downregulated, resulting in the reduction of osteoclast differentiation or increased osteoclast differentiation. When miR-223 expression is extremely low due to the effects of anti-miR-223 oligonucleotides or siRNA, the elevation of IKKα and NFIA expression causes a concomitant downregulation in osteoclast differentiation. This indicates the existence of a switch point in the expression levels of miR-223 that controls the inhibitory and stimulatory effects on osteoclast differentiation. Therefore, identification of this switch point is a prerequisite for the clinical use of miR-223 in treating pathological bone destruction diseases.

It has been found that miRNAs are useful diagnostically and therapeutically in several malignancies [85]. They can be detected, and their concentrations measured, in small peripheral blood samples. In serum, they are stable at room temperature and can withstand freeze-thaw conditions [87]. Therefore, miR-223 represents a promising diagnostic and/or prognostic tool for the treatment of bone diseases that are related to aberrant bone metabolism, such as RA, OA and osteoporosis. Similarly, miR-223 is implicated as a biomarker that can be used to monitor the effectiveness of therapies.

The improved stability and long-lasting effects of synthetic anti-miRNAs make complementarity-based inhibition when given intravenously or locally to specific sites within the cardiovascular compartment, which is an exciting potential treatment modality of the future [88]. Miravirsen, an anti-miRNA of miR-122, can be considered as a representative of this new class of therapeutic agents. It has undergone phase II testing in patients infected with the hepatitis C virus [89]. Down-regulating the expression of miR-223 to extremely low level can either decrease osteoclastogenesis or enhance osteoblast differentiation, an approach that represents a therapeutic strategy for the treatment of bone metabolism disorders which increase bone resorption or decrease bone formation.

There is a number of methods to deliver miR-223 in pathological tissues, for example, augmenting miRNA levels can be used to deliver miRNA through viral-based vectors such as adenoviruses, adeno-associated viruses, and lentiviruses. Also, systemic delivery of siRNAs have been developed and tested by lipid- and polymer-based nanoparticles [90]. Furthermore, microvesicles and apoptotic bodies, which contain circulating miRNAs, can be considered as a therapeutic transport system [91]. However, overexpressing miR-223 is not suitable for treatment of pathological bone destruction diseases, on account of its dual effect in stimulating osteoclast differentiation and inhibiting osteoblast differentiation. Replacing downregulated miRNA or increasing miRNA expression is problematic for a number of reasons, which include their short half-lives and short durations of action [92,93]. The solution is likely to be miRNA mimics designed for specific sites and delivered using novel systems [88].

Given that individual miRNA modulate over 100 target genes, their modulation might reasonably be expected to result in both positive (therapeutic) and negative (pathological) effects. This may also be the case with antisense RNA oligonucleotides (ASOs) against miRNA. Multiple genes acting together in a common pathway can be the target of a single miRNA. An equally important consideration is to determine the appropriate doses to use of ASOs and miRNA mimics. This will require careful evaluation of the pharmacokinetics of differentially modified short oligonucleotides [87]. Therefore, further studies are needed to fully elucidate the modulatory roles of miR-223 targets in bone metabolism, and the association between these targets and miR-223 expression level.

## Abbreviations

**Abbreviations**	**Full Term**
ASO	antisense RNA oligonucleotides
BMMs	bone marrow macrophages
C/EBPα	CCAAT/enhancer binding protein-α
CIA	collagen-induced arthritis
CKD-MBD	chronic kidney disease-mineral and bone disorder
ECT2	epithelial cell-transforming sequence 2
ERKs	extracellular signal regulated kinases
FGFs	fibroblast growth factors
FGFR2	fibroblast growth factor receptor 2
HSP90B1	heat shock protein 90B1
IKKα	inhibitor of nuclear factor κB kinase subunit-α
LVmiR-223T	lentiviral vectors expressing the miR-223 target sequence
MAPK	mitogen-activated protein kinase
M-CSF	macrophage colony-stimulating factor
M-CSFR	macrophage colony-stimulating factor receptor
MEF2C	myocyte-specific enhancer factor 2C
MiRNA	microRNA
MiR-223	microRNA-223
MITF	microphthalmia-associated transcription factor
NFκB	nuclear factor κB
NFIA	nuclear factor 1 A-type
NIK	NF-κB-inducing kinase
NEMO	NF-κB essential modulator
NFATc1	nuclear factor of activated T-cells, cytoplasmic 1
OA	osteoarthritis
PBMCs	peripheral blood mononuclear cells
RA	rheumatoid arthritis
RANK	receptor activator of nuclear factor κB
RANKL	receptor activator of nuclear factor κB ligand
RHOB	rho-related GTP-binding protein RhoB
RUNX2	runt-related transcription factor 2
TRAP	tartrate-resistant acid phosphatase
TRAF2/3	TNF receptor-associated factor 2/3
TGFβ	transforming growth factor beta
VSMCs	vascular smooth muscle cells
3ʹ-UTR	3ʹ-untranslated region
